# Asynchronous generation of oil droplets using a microfluidic flow focusing system

**DOI:** 10.1038/s41598-019-47078-8

**Published:** 2019-07-22

**Authors:** Peter Thurgood, Sara Baratchi, Aram Arash, Elena Pirogova, Aaron R. Jex, Khashayar Khoshmanesh

**Affiliations:** 1 School of Engineering, RMIT University, Melbourne, Australia; 2 School of Health and Biomedical Sciences, RMIT University, Bundoora, Australia; 3 Population Health and Immunity Division, The Walter and Eliza Hall Institute of Medical Research, Parkville, Australia; 4 Faculty of Veterinary and Agricultural Sciences, The University of Melbourne, Parkville, Australia

**Keywords:** Mechanical engineering, Fluid dynamics

## Abstract

Here, we show that long-term exposure of PDMS based microfluidic droplet generation systems to water can reverse their characteristics such that they generate oil-in-water droplets instead of water-in-oil droplets. The competition between two oil columns entering via the two side channels leads to asynchronous generation of oil droplets. We identify various modes of droplet generation, and study the size, gap and generation rate of droplets under different combinations of oil and water pressures. Oil droplets can also be generated using syringe pumps, various oil viscosities, and different combinations of immiscible liquids. We also demonstrate the ability to dynamically change the gap between the oil droplets from a few hundred microns to just a few microns in successive cycles using a latex balloon pressure pump. This method requires no special equipment or chemical treatments, and importantly can be reversed by long-term exposure of the PDMS surfaces to the ambient air.

## Introduction

Flow focusing systems are widely used for the continuous generation of droplets in microfluidics^1–4^. The size, gap and generation rate of droplets can be controlled by varying the viscosity, interfacial tension and flow rates of incoming core and sheath flows^5^. The surface properties of the microfluidic structure can also influence the generation of droplets^6^. For example, the hydrophobicity of PDMS makes it challenging to generate stable oil-in-water droplets^7,8^ as well as generating double emulsions of oil-in-water-in-oil or water-in-oil-in-water droplets^7–9^ in PDMS based droplet generation systems.

Several methods have been developed for the surface modification of PDMS, which can be categorised into surface activation, physical adsorption and chemical modification approaches^10,11^. Surface activation methods rely on oxidation of the PDMS surface, which can be achieved by treatment of the PDMS surface with oxygen plasma^12,13^, ultraviolet/ozone^14^ or the application of acidic solutions containing aggressive components such as hydrogen peroxide^15^. In comparison, physical adsorption methods rely on the functionalisation of the PDMS surface with a thin film of hydrophilic material, which can be achieved by incubation of PDMS structures with non-ionic surfactants^16^ or polymers such as poly(ethylene glycol)^17^ and polyvinyl alcohol^18,19^, layer-by-layer deposition of polyanionic and polycationic films^7,8^ as well as incorporation of non-ionic surfactants^20^ or hydroxy-terminated polymers^21,22^ into the uncured PDMS. Alternatively, chemical modification of PDMS surface can be achieved by silanisation^23^, sol-gel coating^9,24^, chemical vapour deposition^25^ or grafting of hydrophilic polymers^10,26^.

In this work, for the first time, we show that the long-term exposure of PDMS based microfluidic flow focusing systems to water can reversibly modify their droplet generation characteristics such that they can produce oil-in-water droplets instead of water-in-oil droplets. The competition between two oil columns originating from the two side channels leads to the asynchronous generation of oil droplets in water. We explore various modes of asynchronous droplet generation, and investigate the size, gap and generation rate of droplets under various inlet pressures provided by self-sufficient pressure pumps made of latex balloons. We also study the generation of oil droplets using syringe pumps, various oil viscosities, and different immiscible liquids. We demonstrate the ability to rapidly change the gap between the oil droplets in successive cycles. This method does not require any special equipment or chemical treatments, and importantly can be reversed by simply drying the channels with air.

## Results and Discussions

### Changing the wetting properties of the PDMS surface

**Figure 1:**
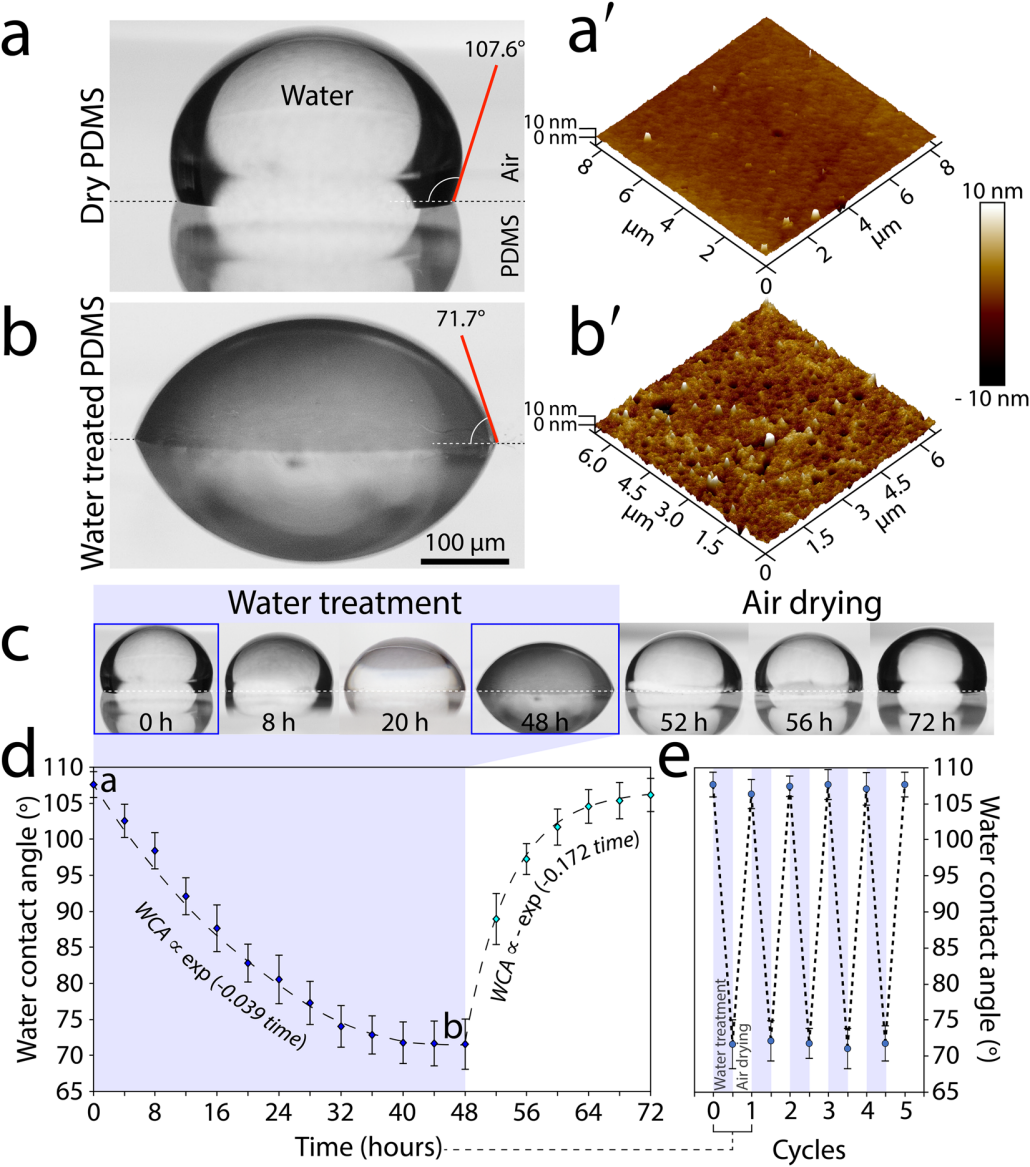
Altering the wettability of PDMS by water treatment: **(a,b)** Water contact angle of dry and water-treated (after 48 hours) PDMS surfaces. (**a**′**-b**′) Surface roughness of dry and water-treated PDMS surfaces obtained by AFM analysis. **(c-d)** Variations in water contact angle as a function of time, showing the ability to reverse the wetting properties of PDMS in repeated cycles. **(e)** Reversible water contact angles over successive cycles. Average ± standard deviation values are based on 5 experimental repeats per each PDMS block, with 3 PDMS blocks being used for each experiment.

While studying microfluidic droplet generation systems we noticed that the wettability of the microfluidic channels, and thus the overall behaviour of the system, can be altered by the long-term treatment of the channels with water. To further investigate this effect, we placed a PDMS slab in a Petri dish filled with DI water for 48 hours. The PDMS slab was then removed from the Petri dishes, and the surface of the PDMS was dried using a cleanroom wipe. A 10 µL droplet of DI water was then placed onto the PDMS surface, and the contact angle between the droplet and the PDMS surface was analysed, as detailed in the Materials and Methods section. The contact angle of the dry PDMS was measured as 107.6 ± 2.6° (Fig. 1a), which is in line with the values reported in the literature^18^. Interestingly, the contact angle of the water-treated PDMS reduced to 71.7 ± 3.3° (Fig. 1b), indicating that the PDMS surface had become hydrophilic.

AFM analysis revealed a notable difference in the surface roughness of the dry and water-treated PDMS slabs. The root-mean-square of the surface roughness were measured as 0.89 nm for the dry PDMS (Fig. 1a′), which increased to 2.44 nm following a 48-hour water treatment (Fig. 1b′). The increased surface roughness is attributed to the gas permeability of the PDMS, which allows the PDMS structure to be saturated with water vapour. This also turns the thick PDMS slab from transparent to cloudy following the 48-hour water treatment (Fig. S1). Our extended experiments indicated that the increased hydrophilicity of the water-treated PDMS reduces the adsorption of immunoglobulin G (IgG) antibody onto the surface (Fig. S2).

In order to capture the dynamic change of the PDMS surface in response to water treatment, we measured the water contact angle in 4-hour intervals over a period of 48 hours (Fig. 1c-left). Our analysis indicated that the water contact angle of water-treated PDMS decreases exponentially over time (*WCA* ∝ exp(−0.039 *time*)) (Fig. 1d). More interestingly, we found that the hydrophobic properties of the PDMS surface can be recovered by simply exposing the surface to the ambient air (Fig. 1c-right). Our dynamic analysis indicated that the water contact angle of air-drying PDMS increases exponentially over time (*WCA* ∝ −exp(−0.172 *time*)) (Fig. 1d). We further investigated the effectiveness of this method for the reversible change of surface wetting properties in successive cycles (Fig. 1e).

Using our method, the wetting properties of PDMS surfaces can be dynamically changed by simply varying the amount of time that the surface is exposed to water. Our proof-of-concept experiments indicated the suitability of this method for changing the affinity of the surface to oil droplets or the antibodies suspended in the solution. Compared to existing reversible surface modification methods, which require external stimuli such as voltage^27^, electric field^28^, magnetic field^29^, temperature^30^ or UV irradiation^23^, and importantly require extensive surface preparation by incorporation of micro/nano structures^29^, porous films^28,30^ or chemical treatments^23,27^, our reversible surface modification method can be realised using ordinary PDMS microfluidic structures and water treatment, and thus is easier to implement and importantly is more compatible to bio-microfluidic experiments^31,32^.

### Asynchronous generation of oil droplets

**Figure 2:**
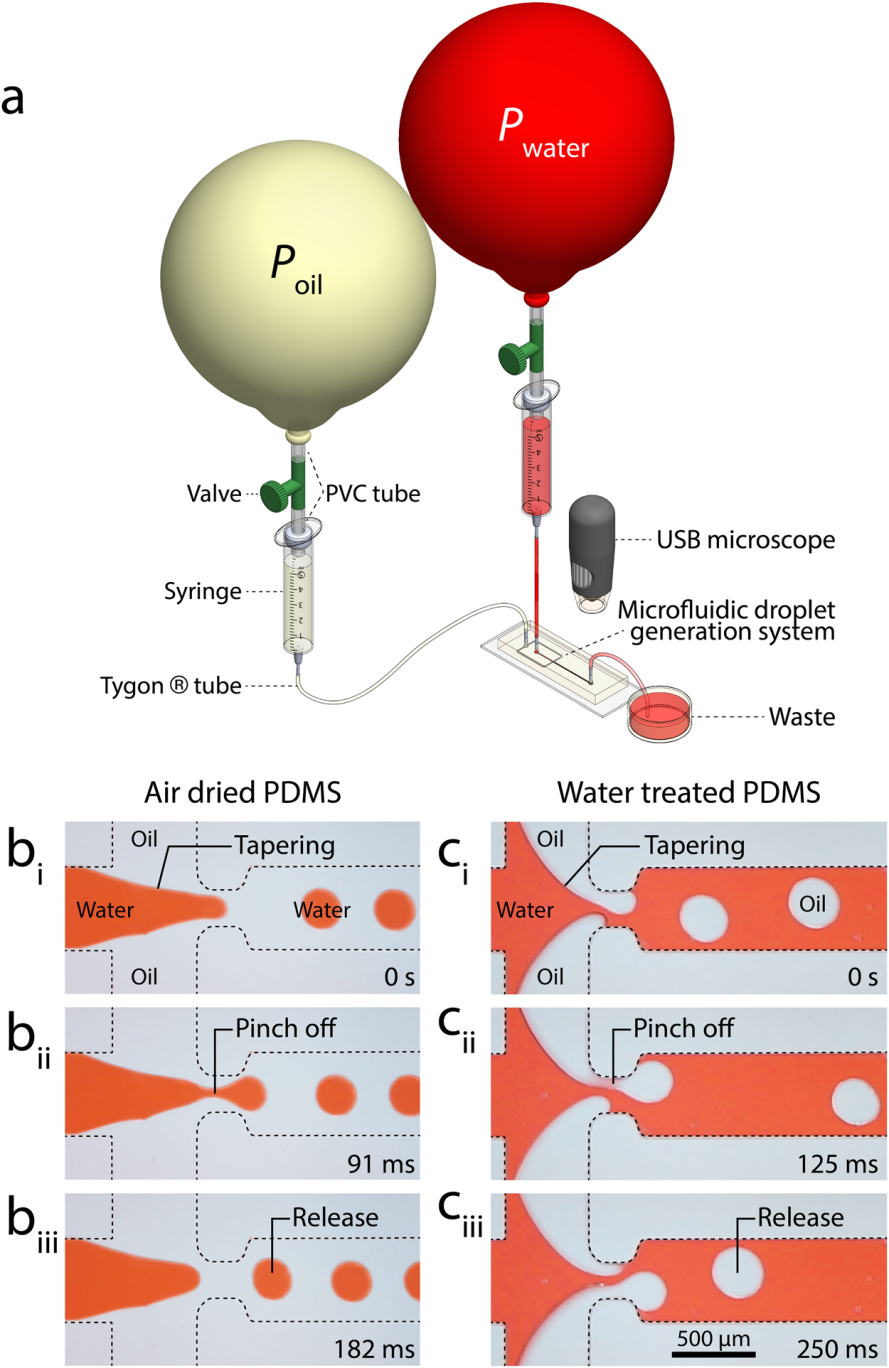
Comparison of droplet generation within the *air-dried* and *water-treated* microfluidic structures: **(a)** Experimental setup consisting of a flow focusing structure coupled with two balloon-based pressure pumps to drive red-stained water and mineral oil. **(b)** Generation of water droplets in oil within the *air-dried* channel during tapering, pinch-off, and release steps. **(c)** Asynchronous generation of oil droplets in water within the *water-treated* channel during tapering, asynchronous advancement, pinch-off, and release steps.

A series of experiments were conducted to explore the influence of PDMS surface treatment on microfluidic droplet generation. First, we investigated the droplet generation characteristics of the *air-dried* PDMS structure. To do so, we injected red stained DI water mixed with surfactant (5% w/w TWEEN®20, Sigma-Aldrich) into the middle inlet channel while injected mineral oil into the two side inlet channels of the microfluidic system (Fig. 2a). The contact angle of the carrier phase solution is presented in Fig. S3. The geometric details of the microfluidic structure are shown in Fig. S4.

Latex balloons were used as the source of pressure for driving the liquids through the microfluidic system. In this regard, the balloon acts as a low-cost, self-sufficient pressure pump. Unlike a syringe pump-driven system, in which the flow rate set by the user determines the pressure gradient across the microfluidic system (*Q*_set_ → ∆*P*), in a pressure pump-driven system the pressure drop set by the user determines the flow rate through the microfluidic system (∆*P*_set_ → *Q*). For example, in our case, the inlet pressure equals the internal pressure of the balloon and the outlet pressure equals the ambient pressure, providing ${\rm{\Delta }}{P}_{set}={P}_{{\rm{balloon}}}-{P}_{\infty }={P}_{{\rm{inflation}}}$. The suitability of pressure pump-driven systems for the continuous generation of droplets has been demonstrated in several works^33–36^.

The pressure of the balloons depended on the thickness of the balloon shell as well as the circumference of the inflated balloon, as detailed in our recent work^37^. We coupled a pair of double-layered latex balloons with a circumference of 77.5 and 73.5 cm (*P*_water_ ~ 4.75 kPa and *P*_oil_ ~ 4.1 kPa) to the water and oil inlets. This led to the generation of water droplets in oil similar to a conventional microfluidic flow focusing droplet generation system^37^ (Movie S1).

A closer look at the static images revealed that the oil advanced along the sidewalls of the water inlet channel. The shear stress at the water-oil interface tapered the water column when advancing toward the orifice (Fig. 2b_i_). While passing through the orifice, the excessive shear stress at the oil-water interface caused the advancing water column to break into water droplets, which is known as ‘pinch-off’ (Fig. 2b_ii_). The droplets were released into the outlet channel with an average diameter of 252 µm at 380 droplets/min (Fig. 2b_iii_).

Next, we investigated the droplet generation characteristics of the *water-treated* PDMS structure. To do so, we coupled a pair of single-layered latex balloons with a circumference of 64.0 and 77.2 cm (*P*_water_ ~ 1.59 kPa and *P*_oil_ ~ 2.38 kPa) to the water and oil inlets. Interestingly, this led to the generation of oil droplets in water (Movie S2).

In this case, the water advanced along the sidewalls of the oil inlet channels (Figure S5). This caused the oil columns to become thinner, forming two tapered columns on either side of the orifice. These two tapered columns advanced asynchronously through the orifice (Fig. 2c_i_). The competition between the two oil columns intensified the shear stress at the interface of oil-water, causing the advancing oil columns to break into oil droplets in an asynchronous manner (Fig. 2c_ii_). The oil droplets were released into the outlet channel with an average diameter of 303 µm at 258 droplets/min (Fig. 2c_iii_). This alternating generation of droplets resembles the vortex shedding phenomenon occurring at high Reynolds numbers at the downstream of circular structures^38^.

The hydrophilic properties of the water-treated PDMS channel could be maintained as long as the channel remained wet. The process of surface modification was reversible and followed similar dynamics to those presented in Fig. 1c. The PDMS channel became hydrophilic by applying DI water through the microfluidic structure at a low flow rate of ~15 µL/min for 48 hours. To recover the PDMS hydrophobic properties, the microfluidic structure was washed, emptied by applying suction, and exposed to ambient air for 24 hours to dry.

### Identification of various modes of asynchronous oil droplets

**Figure 3:**
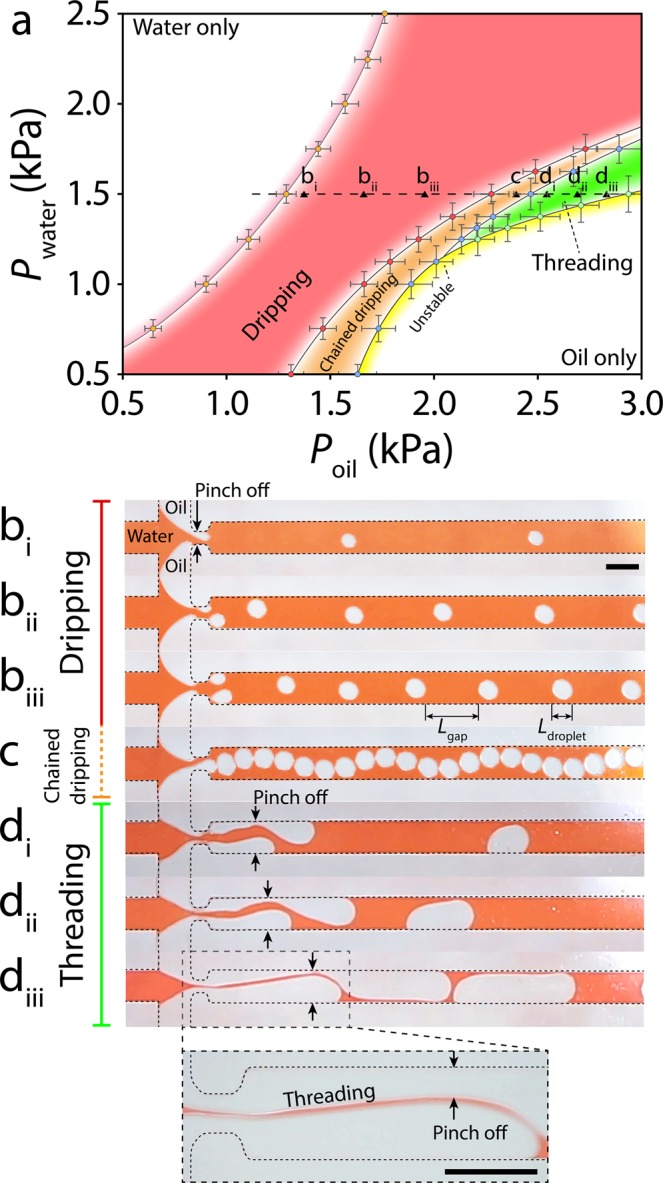
Characterisation of asynchronous oil droplets at various combinations of oil and water pressure: **(a)** Droplet generation map, showing the existence of ‘dripping’, ‘chained dripping’, ‘threading’ and ‘unstable’ modes. Average ± standard deviation values are based on 10–50 droplets per each experiment according to the stability of droplets, with 4 experimental repeats for each device, and 3 devices being used for each experiment. **(b)** ‘Dripping’ mode corresponding to the asynchronous generation of droplets at the orifice. **(c)** ‘Chained dripping’ mode corresponding to generation of droplets that are separated by a thin layer of water. **(d)** ‘Threading’ mode corresponding to the advancement of oil columns through the orifice and the asynchronous generation of droplets along the sidewalls. Increase of oil pressure leads to the formation of long droplets that are separated by a thin thread of water. Scale bars are 500 µm.

Next, we investigated the characteristics of asynchronous oil droplets under various combinations of oil and water pressures. To do so, we varied the water pressure from 0.5 to 2.5 kPa in 0.25 kPa intervals by changing the circumference of the water balloon. These pressure lines are shown as dashed lines in Fig. 3a. For each water pressure, we varied the oil pressure in various intervals as small as 0.05 kPa by simply raising the oil containing syringe. These experimental points are presented as dark triangles in Fig. 3a. To highlight the diverse dynamics of the oil droplets, we focus on a specific set of experiments conducted at *P*_*water*_ = 1.5 kPa, as discussed below and presented in Movie S3:

#### Water only

This mode occurred at low oil pressures within the range of *P*_*oil*_ < 1.3 kPa. The outlet channel was filled with water with no oil droplets generated. It is notable that this mode could only be achieved using pressure pumps.

#### Dripping

This mode occurred at moderate oil pressures in the range of 1.3 < *P*_*oil*_ < 2.2 kPa. The competition between the opposite oil columns led to the asynchronous generation of oil droplets at the orifice, as comprehensively discussed in Fig. 2c. Increase in oil pressure increased the size of oil droplets while decreasing the gap between them (Fig. 3b_i_–b_iii_).

#### Chained dripping

This mode occurred at high oil pressures within the range of 2.2 < *P*_*oil*_ < 2.4 kPa. The high oil pressure led to generation of larger droplets that were only separated by a thin layer of water. The released droplets bounced between the opposite sidewalls, which caused the droplet chain to follow a sinusoidal pattern along the outlet channel (Fig. 3c). This sinusoidal pattern weakened as the droplets became larger toward the end of this pressure range.

#### Threading

This mode occurred at higher oil pressures in the range of 2.4 < *P*_*oil*_ < 2.8 kPa. The high pressure of competing oil columns facilitated their passage through the orifice. The oil columns advanced along the sidewalls of the outlet channel before being broken into droplets due to the expansion of the trailing oil column (Fig. 3d_i_). The released droplets slid along the sidewalls. Increase of oil pressure increased the size of oil droplets while decreasing the gap between them (Fig. 3d_ii_–d_iii_).

#### Unstable

This mode occurred at oil pressures in the range of *P*_*oil*_ > 2.4 kPa. The expansion of oil droplets along with the reduced gap between them induced a large pressure drop along the outlet channel that in turn caused the oil droplets to collapse only after 60 s, following which the outlet channel was filled with oil. The transition into the unstable mode is further detailed in Movie S4.

### Characterization of asynchronous oil droplets

**Figure 4:**
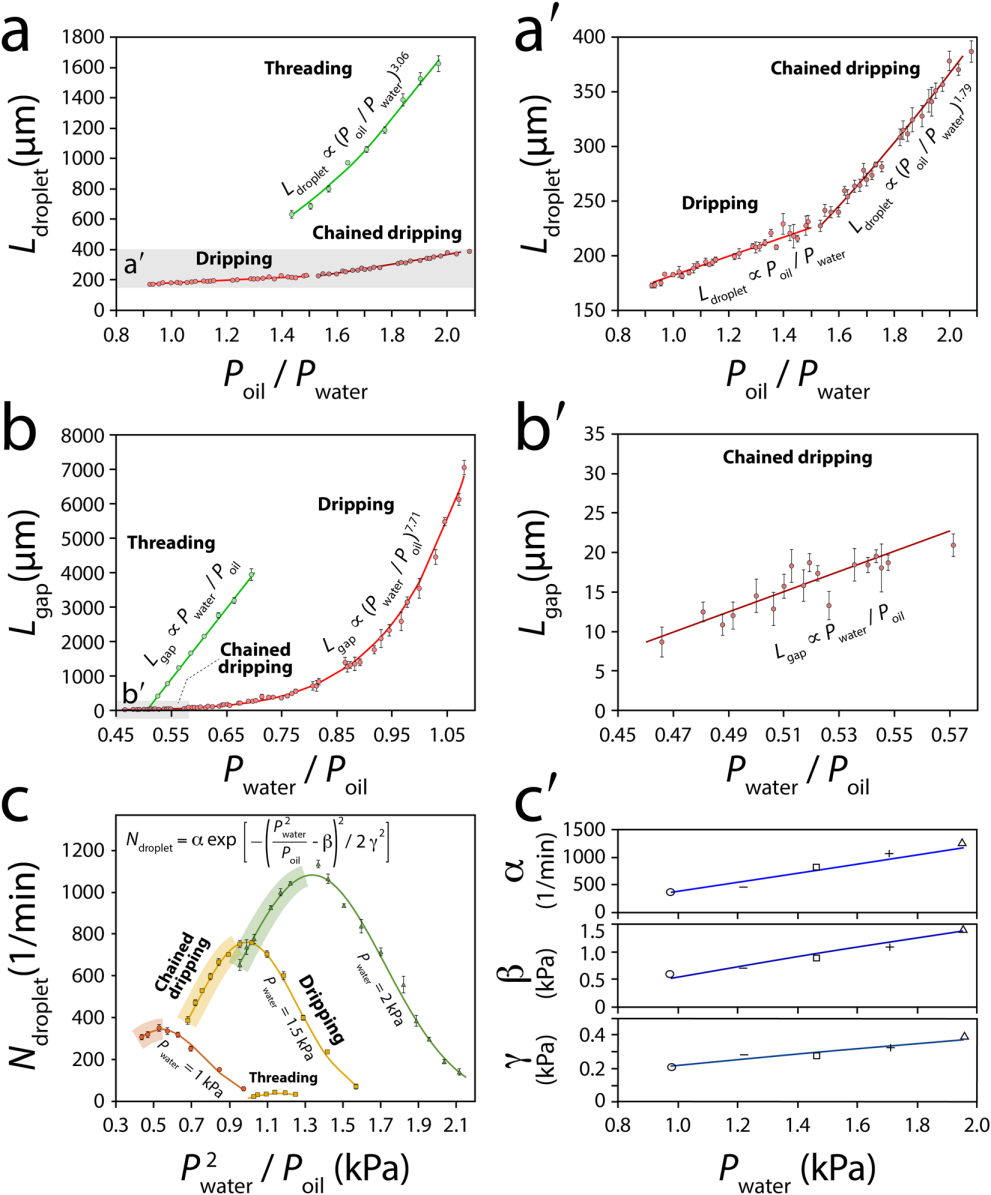
Characterisation of asynchronous oil droplets at various oil and water pressures: **(a-a**′**)** Variation in droplet size against ${P}_{oil}/{P}_{water}$, **(b-b**′**)** Variation in droplet gap against ${P}_{water}/{P}_{oil}$, **(c)** Variation in droplet generation rate against ${P}_{water}^{2}/{P}_{oil}$, (**c**′) Variation in the variance (*α*), mean (*β*) and standard deviation (*γ*) coefficients used in the Gaussian distribution function against ${P}_{water}$. Average ± standard deviation values are based on 10–50 droplets per each experiment according to the stability of droplets, with 4 experimental repeats for each device, and 3 devices being used for each experiment.

Next, we characterised the size, gap and generation rate of asynchronous oil droplets generated under ‘dripping’, ‘chained dripping’, and ‘threading’ modes (Fig. 4).

In general, the increase of oil pressure or decrease of water pressure led to the expansion of oil droplets. To facilitate the comparison of experimental results, which have been conducted in various oil and water pressures, we investigated the variations of droplet size against the oil to water pressure ratio (*L*_*droplet*_ *vs*. *P*_*oil*_/*P*_*water*_). The droplets experienced a linear expansion in the ‘dripping’ mode: *L*_*droplet*_ ∝ *P*_*oil*_/*P*_*water*_ but a non-linear expansion in the ‘chained dripping’ mode: *L*_*droplet*_ ∝ (*P*_*oil*_/*P*_*water*_)^1.79^ (Fig. 4a-a′). The non-linear expansion of droplets was intensified in the ‘threading’ mode: *L*_*droplet*_ ∝ (*P*_*oil*_/*P*_*water*_)^3.06^.

Likewise, an increase in oil pressure or decrease in water pressure reduced the gap between the successive droplets. Thus, we investigated the variations of droplet gap against the water to oil pressure ratio (${L}_{gap}\,vs.{P}_{water}/{P}_{oil}$). The droplets experienced a sharp, non-linear gap reduction in the ‘dripping’ mode: ${L}_{gap}\propto {({P}_{water}/{P}_{oil})}^{7.71}$ but a slow, linear gap reduction in the ‘chained dripping’ mode: *L*_*gap*_ ∝ (*P*_*water*_/*P*_*oil*_) (Fig. 4b-b′). A sharp yet linear gap reduction was observed in ‘threading’ mode.

Our extensive experiments indicated that increase of water pressure along with reduction of droplet size could increase the generation rate of droplets: *N*_*droplet*_ ∝ *P*_*water*_ and *N*_*droplet*_ ∝ (*P*_*oil*_/*P*_*water*_)^−1^. To accommodate both parameters, we investigated the variations of droplet generation rate against the water pressure square to oil pressure ratio (${N}_{droplet}\,vs.{P}_{water}^{2}/{P}_{oil}$). Under a constant water pressure, the droplet generation rate followed a Gaussian distribution: ${N}_{droplet}=\alpha \,\exp [-{(\frac{{P}_{water}^{2}}{{P}_{oil}}-{\rm{\beta }})}^{2}/2{\gamma }^{2}]$ across the ‘dripping’ and ‘chained dripping’ modes (Fig. 4c). Increase of water pressure led to linear increase of the variance, mean and standard deviation coefficients, presented as *α*, *β* and *γ* in the Gaussian distribution function (Fig. 4c′). A Gaussian distribution curve was also observed in the ‘threading’ mode (Fig. 4c), as further detailed in Fig. S6.

### Studying asynchronous oil droplets under various operation conditions

#### Orifice width

We investigated the effect of the orifice width on droplet generation. To do this, we reduced the orifice width from 200 to 75 µm. The additional pressure drop caused by the narrowed orifice hindered the advancement of competing oil columns through the orifice, which eliminated the ‘threading’ mode and led to the dominance of ‘dripping’ mode across the entire pressure range. This allowed for the generation of a wide variety of droplet sizes (Movie S5).

#### Syringe pump

We conducted further experiments to test whether the oil droplets can be generated using displacement pumps in the same manner as pressure pumps. To do so, we used a pair of syringe pumps (Pico Plus, Harvard Apparatus) to apply water and oil. Our experiments indicated the ability to generate asynchronous oil droplets in ‘dripping’, ‘chained dripping’, and ‘threading’ modes using syringe pumps. However, increasing the flow rate of oil caused the oil columns to flow along the sidewalls of the outlet channel without being broken into droplets, converting the droplet generator into a flow focusing system (Figures S7 and S8** + **Movie S6).

#### Oil viscosity

We also investigated the effect of oil viscosity on droplet generation. The oil viscosity was increased by mixing two mineral oils (RTM8 oil, ${\mu }_{{\rm{RTM}}8}$* = *10.37 mPa.s and RTM13 oil, ${\mu }_{{\rm{RTM}}13}$= 75.19 mPa.s, Sigma-Aldrich) at various volumetric ratios. The increase of oil viscosity reduced the flow rate of oil entering into the droplet generation system (${Q}_{oil}\propto {\mu }_{oil}^{-1}$), which in turn decreased the droplet size while increased the gap between the droplets (Fig. S9** + **Movie S7).

#### Other immiscible liquid pairs

We conducted additional experiments to examine the versatility of our system when applying various combinations of carrier and discrete liquids. To do so, first, we demonstrated the ability for generating asynchronous air bubbles, which was achieved by applying air into the two side channels (Movie S8). More interestingly, we demonstrated the ability for co-generating asynchronous air bubble/oil droplets (Movie S8). This was made possible when a small pocket of air was trapped in one of the side channels, enabling the air and oil columns to enter via the opposite side channels. We also presented the ability for generating asynchronous oil droplets using isopropanol (in the absence of TWEEN® 20) as the carrier fluid (Movie S9). Interestingly, the oil droplets slid along the sidewalls of the outlet channel.

#### Generation of small oil droplets

Our extended experiments indicated the ability for generating droplets smaller than the orifice size when operating at low ${P}_{oil}/{P}_{water}$ ratios (Fig. 4a-a′). For example, operating a microfluidic system with an orifice size of 200 µm at ${P}_{water}$ = 2.0 kPa and ${P}_{oil}$ = 2.1 kPa, enabled us to generate droplets with an average size of 159 µm in the dripping mode, which was 20.5% smaller than the orifice size (Movie S10). This is attributed to the narrowing of the orifice due to the presence of competing oil columns.

#### Long-term droplet generation

Our experiments proved the ability for the asynchronous generation of droplets over extended periods of time (>8 hours). This was achieved by coupling two latex balloons with a circumference of 65 cm to 30 mL syringes for driving oil and water through the microfluidic structure. The system operated stably with the size of oil droplets being consistent during the long-term experiments (Fig. S10).

#### Effect of plasma treatment

We conducted two control experiments to prove that the asynchronous generation of oil droplets does not rely on the surface oxidation caused by plasma treatment. The first experiment included filling the channels with DI water 24 hours after the plasma treatment, long after the PDMS had recovered its hydrophobicity^12^. The second experiment included using a mechanical clamp (Fig. S11) to stop leakage while avoiding plasma treatment prior to filling the channels with DI water. We conducted this experiment particularly, as extended plasma treatment followed by storage of DI water in the channel has been shown to extend the hydrophilicity of PDMS for a few weeks^13,39^. Both control experiments indicated that the asynchronous generation of oil droplets is purely due to the long-term exposure of the PDMS to DI water.

### Dynamic spacing between droplets

**Figure 5:**
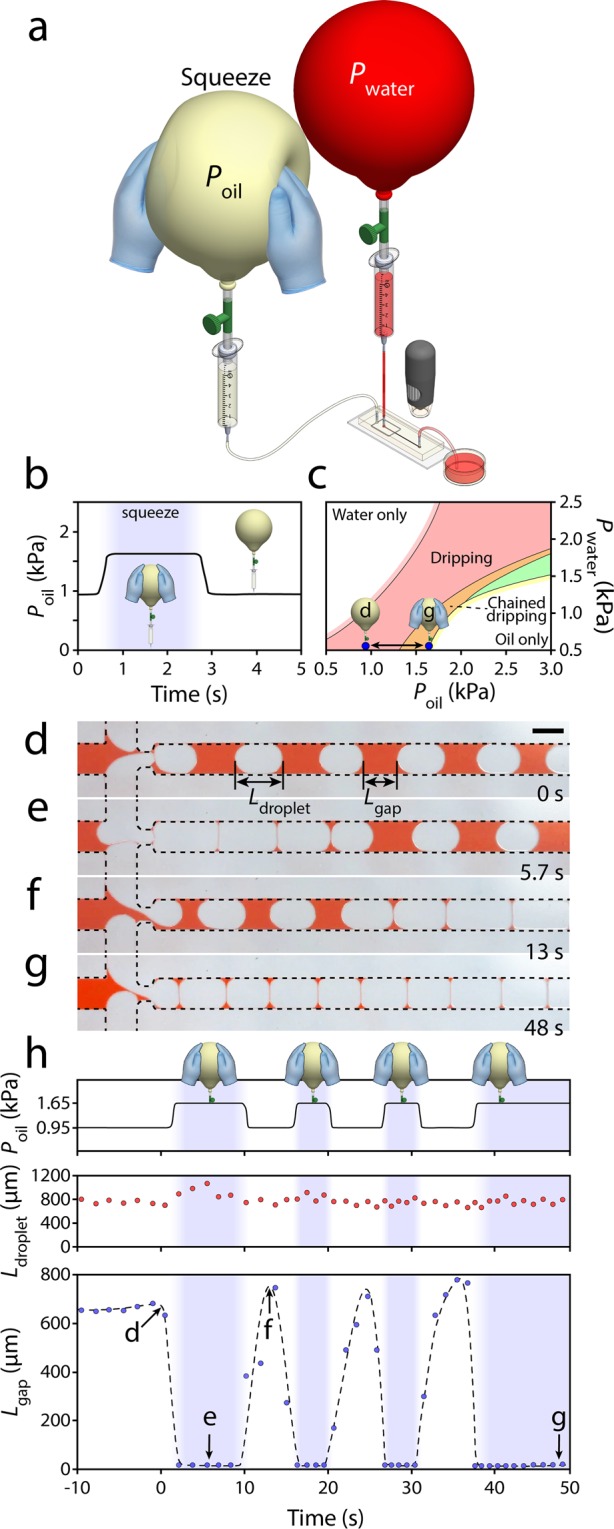
Dynamic spacing between generated droplets enabled by the manual compression of a balloon pump. **(a)** Experimental setup consisting of two balloon pumps coupled to a microfluidic flow focusing system, **(b)** Variation in balloon internal pressure in response to manual squeezing, **(c)** Dynamic transition between ‘dripping’ and ‘unstable’ droplet generation modes following the manual squeezing of the oil balloon. (**d**–**g**) The ability to change the gap between the generated droplets almost instantly, and **(h)** Variations in oil balloon pressure, droplet size and droplet gap in four successive squeezing cycles. Scale bar is 500 µm.

Next, we studied asynchronous generation of oil droplets under dynamic conditions. The hyper-elastic structure of the latex balloon allowed us to dynamically change the internal pressure of the balloon via manual squeezing (Fig. 5a), as comprehensively described in our previous work^37^.

The oil and water balloons were inflated to a circumference of 65 cm (~1.6 kPa). The effective pumping pressure of the water and oil balloons was reduced to 0.5 and 0.95 kPa, respectively, by lowering the coupled syringes by 11.25 and 6.65 cm. Manual squeezing of the oil balloon by approximately ~5 cm from both sides increased the effective pumping pressure to ~1.65 kPa. The pressure increased almost instantaneously and could be maintained as long as the balloon was squeezed (Fig. 5b). This allowed us to switch from one droplet generation mode to another rapidly (Fig. 5c).

Prior to squeezing of the oil balloon, the droplets were generated in the ‘dripping’ mode. The average size and gap of droplets were measured as *L*_droplet_ = 757 µm and *L*_gap_ = 640 µm, respectively (Fig. 5d). Following the squeezing of the oil balloon, the droplets were generated in an ‘unstable’ dripping mode. The average droplet size increased to *L*_droplet_ = 788 µm while the average gap decreased to *L*_gap_ < 10 µm (Fig. 5e). In this mode, the droplets disordered and would eventually collapse after ~270 s (Movie S11). Releasing the oil balloon increased the droplet gap and returned the droplets into a stable ‘dripping’ mode (Fig. 5f). The system could be switched between the ‘dripping’ and ‘unstable’ modes very smoothly and rapidly with only 1 droplet delay (Fig. 5g** + **Movie S12). This is significant, as the ‘unstable’ mode could only be operated for a short period (as reflected by its name) due to merging of neighbouring droplets. Switching between the ‘dripping’ and ‘unstable’ modes enabled us to generate a limited number of droplets (<10) in ‘unstable’ mode in repeated cycles without collapsing the system. This, in turn, varied the oil pressure, droplet size and droplet gap in four successive squeezing cycles (Fig. 5h), as further detailed in Fig. S12.

## Conclusions

In summary, we showed the long-term exposure of PDMS to water as an effective means for increasing their hydrophilicity. This simple method was used for converting a flow focusing system designated for generating water droplets into a double T-junction system^40^ capable of asynchronously generating oil droplets. The characteristics of the system were governed by the pressure ratio of oil to water, based on which the droplets could be generated in ‘dripping’, ‘chained dripping’, ‘threading’ and ‘unstable’ modes. Asynchronous droplets could be generated using both pressure and syringe pumps as well as relatively high viscosity oils. We also demonstrated the ability to vary the gap between the successive droplets from a few hundred microns to a few microns in successive, rapid cycles. The hydrophobicity of the PDMS surface could be reversed by drying the channels in ambient air.

The novel method for asynchronous generation of droplets, presented here, creates opportunities for making soft, reconfigurable electronic devices. This can be achieved by using conductive liquids such as gallium-based liquid metal alloys^41^ as the carrier phase and non-conductive oils as the dispersed phase. Dynamic changing of the size and gap of oil droplets leads to dynamic change of the conductivity and thus, the characteristics of the device (Fig. S13a). Also, generation of droplets made of UV curable oils^42^ provides opportunities for fabrication of highly complex microfluidic structures within the mixing channel. The morphology of such structures can be varied based on the geometry of droplets, turning a lithographically made channel with a rectangular cross section into a complex conduit (Fig. S13b). Furthermore, the generated oil droplets can be collected, pattered and utilised as soft barriers (Fig. S13c). This provides unprecedented opportunities for making organ-on-chip systems incorporating soft, reconfigurable structures for studying various diseases^43^ and worm-on-chip platforms suitable for studying multi-cellular organisms such as *Caenorhabditis elegans* worms^44^. Oil droplets can be displaced or removed enabling a fully reconfigurable system. Oil droplets also lead to local changes in the flow velocity, which be used for producing customised shear stress profiles^31,45^ or even inertial manipulation of microscale particles^46^.

## Materials and Methods

### Measurement of droplet contact angle

The contact angle between the water droplet and the PDMS surface was measured by placing a 10 µL droplet of deionised water on the surface of PDMS slabs (Sylgard® 184, Dowsil, cured at 120 °C for 20 min) using a pipette (Eppendorf). Contact angle images were taken using a Canon 6D camera coupled with a Canon 100 mm macro lens. The contact angles were then measured using ImageJ.

### Experimental setup

The experimental setup consisted of self-sufficient pressure pumps made of commercially available latex balloons, as recently reported^37^, coupled to a microfluidic flow focusing system made of PDMS. The microfluidic system was fabricated by patterning 80 µm thick SU-8 structures on 4-inch silicon wafers using a MLA150 Maskless Aligner (Heidelberg Instruments, Germany). PDMS base and curing agent (Sylgard® 184, Dowsil) were mixed at a weight ratio of 10:1 and degassed under vacuum before being poured onto the silicon master placed in a 110 mm glass Petri dish. The PDMS structures were cured at 120 °C for 20 min using a vacuum oven (Thermo Fisher Scientific). The PDMS structures were then peeled off the master, cut into the desired dimensions using a scalpel, and liquid interfaces were punched using biopsy punches (0.75 mm, Harris Uni-Core) before being permanently plasma bonded (Harrick Plasma PDC-002) to glass microscope slides (Thermo Scientific, 76 × 26 × 1 mm). The microfluidic system was interfaced with the pressure pumps using Tygon® tubing (OD = 1.5 mm, ID = 0.5 mm, *L*_inlet_ = 20 cm, *L*_outlet_ = 10 cm, Sigma-Aldrich).

The balloon-based pressure pumps consisted of helium quality latex balloons (Artwrap, Australia, 25 cm) coupled to plastic syringes (5 mL Braun, Germany) via short sections of PVC aquarium tubing (OD = 6 mm, ID = 4 mm) with a 2-way plastic air valve (Aqua One, Australia)^37^. The pressure pumps were interfaced with a microfluidic flow focusing system. The balloon inflation pressure defined as ${P}_{{\rm{inflation}}}={P}_{{\rm{balloon}}}-{P}_{\infty }$ was measured using a digital manometer (AHJ Systems, 8205) (Fig. S14).

The width of the oil and water inlet channels and the outlet channel were set to 500 µm. The inlet and outlet channel were interconnected via an orifice with a width and of 200 µm (Fig. S4). The height of the system was set to 80 µm.

Mineral oil (RTM8, Sigma-Aldrich, *μ*_oil_ = 10.37 mPa.s) and deionised water mixed with polysorbate (5% w/w, TWEEN® 20, Sigma-Aldrich) were used as a pair of immiscible liquids for generating droplets.

### Atomic force microscopy (AFM)

AFM imaging was performed to examine the topography of PDMS surfaces before and after surface treatment. This was conducted using an AFM system (Dimension Icon, Bruker) operated in contact mode, and interfaced with NanoScope analysis software (Bruker Corporation).

### Protein adsorption

PDMS surfaces were incubated with different concentrations of goat IgG directly labelled with Alexa 488 (Thermofisher scientific) for 2 hours at 37 °C. This was followed by three washes to remove unbounded proteins. Imaging was performed using a 488 nm laser and Nikon A1 laser scanning confocal microscope. Fluorescence emission was detected using a photomultiplier tube following a 525/50 nm band-pass filter using a 10 × objective.

### Statistical analysis

Statistical analysis was performed to characterise the contact angle of droplets, antibody adsorption and behaviour of droplets using dry and water-treated PDMS surfaces, as summarised below.

Contact angle experiments were performed by inserting 5 droplets onto each PDMS block and using 3 PDMS blocks, thus providing us with 15 sets of results for each experimental condition. Experiments were conducted in parallel for 72 hours. The contact angles were measured using ImageJ software, and reported as average ± standard deviation.

Antibody adsorption experiments were performed by inserting 3 droplets onto each PDMS block and using 3 PDMS blocks at each antibody concentration, providing us with 9 sets of results for each case. Fluorescent images were taken using a Nikon A1 laser scanning confocal microscope. Antibody adsorption was measured using NIS-Element software (Nikon), and reported as average ± standard deviation.

Droplet generation experiments were repeated 4 times using 3 microfluidic structures, thus providing 12 sets of results for each experimental condition. Droplets were imaged using a USB-microscope and analysed using ImageJ software. The size, gap and generation rate of droplets were measured, and reported as average ± standard deviation.

## Supplementary information

Supplementary Information

Movie S1

Movie S2

Movie S3

Movie S4

Movie S5

Movie S6

Movie S7

Movie S8

Movie S9

Movie S10

Movie S11

Movie S12

## Data Availability

All data generated or analysed during this study are included in this published article (and its Supplementary Information Files).
