# Supplementary material for: Asynchronous generation of oil droplets using a microfluidic flow focusing system
Source: Sci Rep. 2019 Jul 22;9:10600. doi: 10.1038/s41598-019-47078-8 (PMC6646804; doi:10.1038/s41598-019-47078-8)
Supplement: Supplementary file 1 — Supplementary Information [file 41598_2019_47078_MOESM1_ESM.pdf]

Supplementary Information

Asynchronous generation of oil droplets using a microfluidic flow focusing system

Peter Thurgood [1,†], Sara Baratchi [2], Aram Arash [1],
Elena Pirogova [1], Aaron R. Jex [3,4], Khashayar Khoshmanesh [1,†]

[1] School of Engineering, RMIT University, Melbourne, Australia
[2] School of Health and Biomedical Sciences, RMIT University, Bundoora, Australia
[3] Population Health and Immunity Division, The Walter and Eliza Hall Institute of Medical Research, Parkville, Australia
[4] Faculty of Veterinary and Agricultural Sciences, The University of Melbourne, Parkville, Australia

† Corresponding authors:
Peter Thurgood: peter.thurgood@rmit.edu.au
Khashayar Khoshmanesh: Khashayar.khoshmanesh@rmit.edu.au

Supplementary Information S1: Visual comparison of air dried and water-treated PDMS blocks

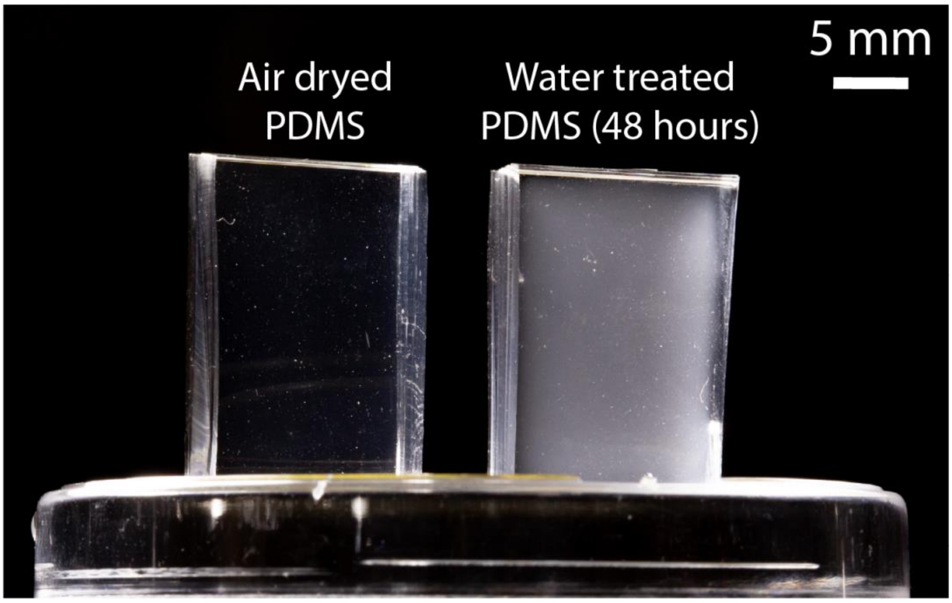

Figure S1: Visual comparison of air dried and DI treated PDMS blocks. The air dried PDMS is transparent whereas the water-treated PDMS is cloudy due to uptake of water vapour. PDMS slabs = 20 mm × 15 mm × 10 mm (height × width × thickness).

Supplementary Information S2: Comparison of antibody adsorption on dry and water-treated PDMS surfaces

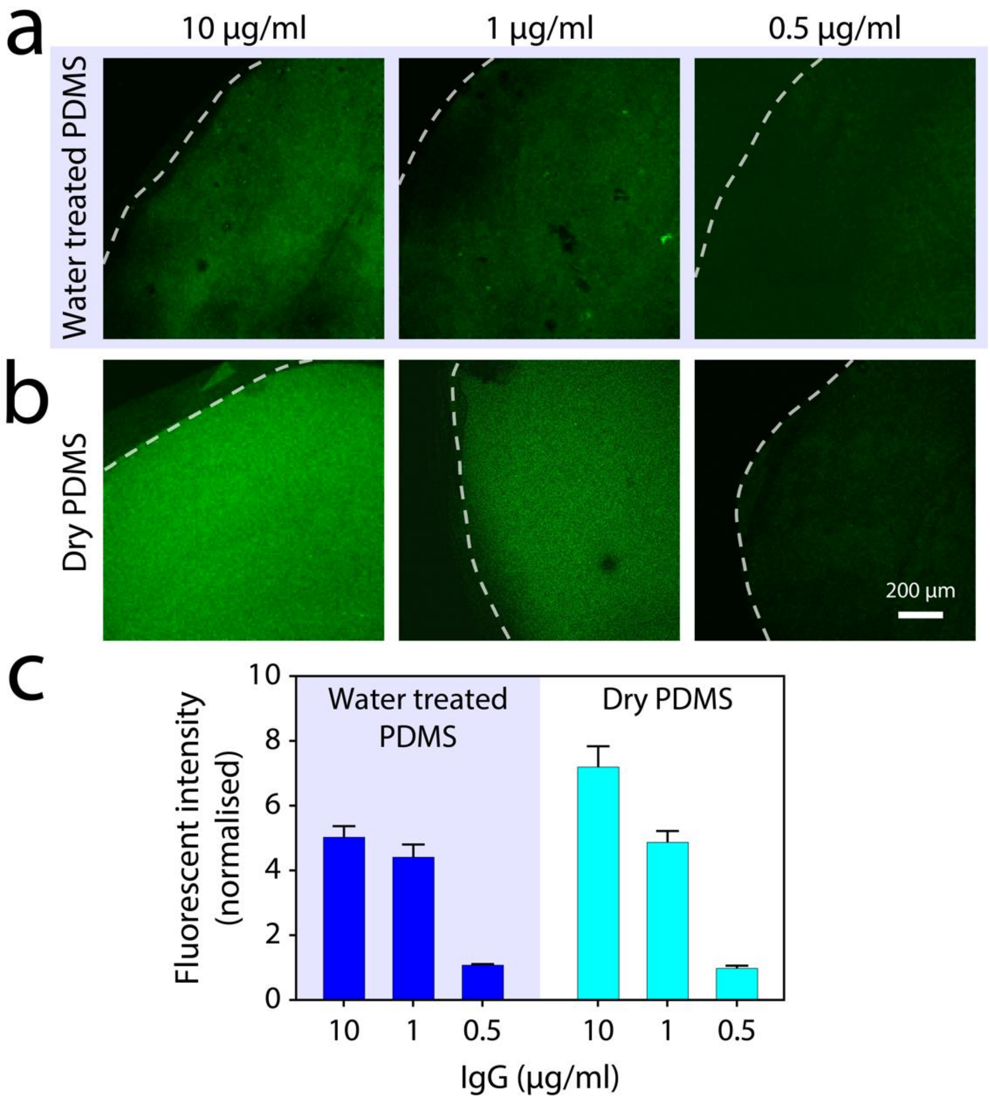

Figure S2: Adsorption of immunoglobulin G (IgG) antibody onto water-treated PDMS (following a 48-hour treatment) and dry PDMS surfaces: **(a-b)** Fluorescent images at various concentrations of IgG with the dashed lines showing the edge of droplet. **(c)** Normalised fluorescent intensities. Average ± standard deviation values are based on 3 experimental repeats for each PDMS block, with 3 PDMS blocks being used in each experiment.

Supplementary Information S3: The contact angle of a solution of DI water mixed with 5% w/w TWEEN20® on dry and water treated PDMS surfaces

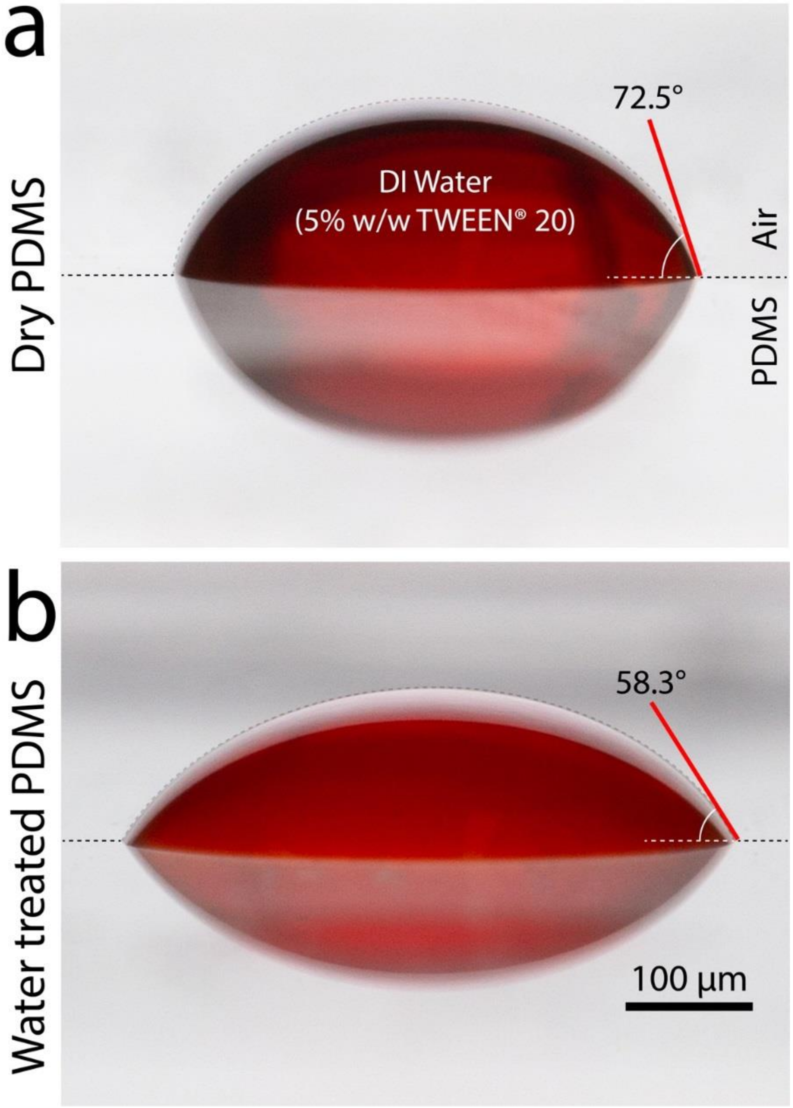

Figure S3: The contact angle of a solution of DI water stained with red food dye and mixed with 5% w/w TWEEN20® (the same solution used for generation of droplets) on dry and water treated PDMS surfaces. **(a)** Dry PDMS surface. **(b)** Water-treated (after 48 hours) PDMS surface.

Supplementary Information S4: Details of microfluidic flow focusing systems used for droplet generation

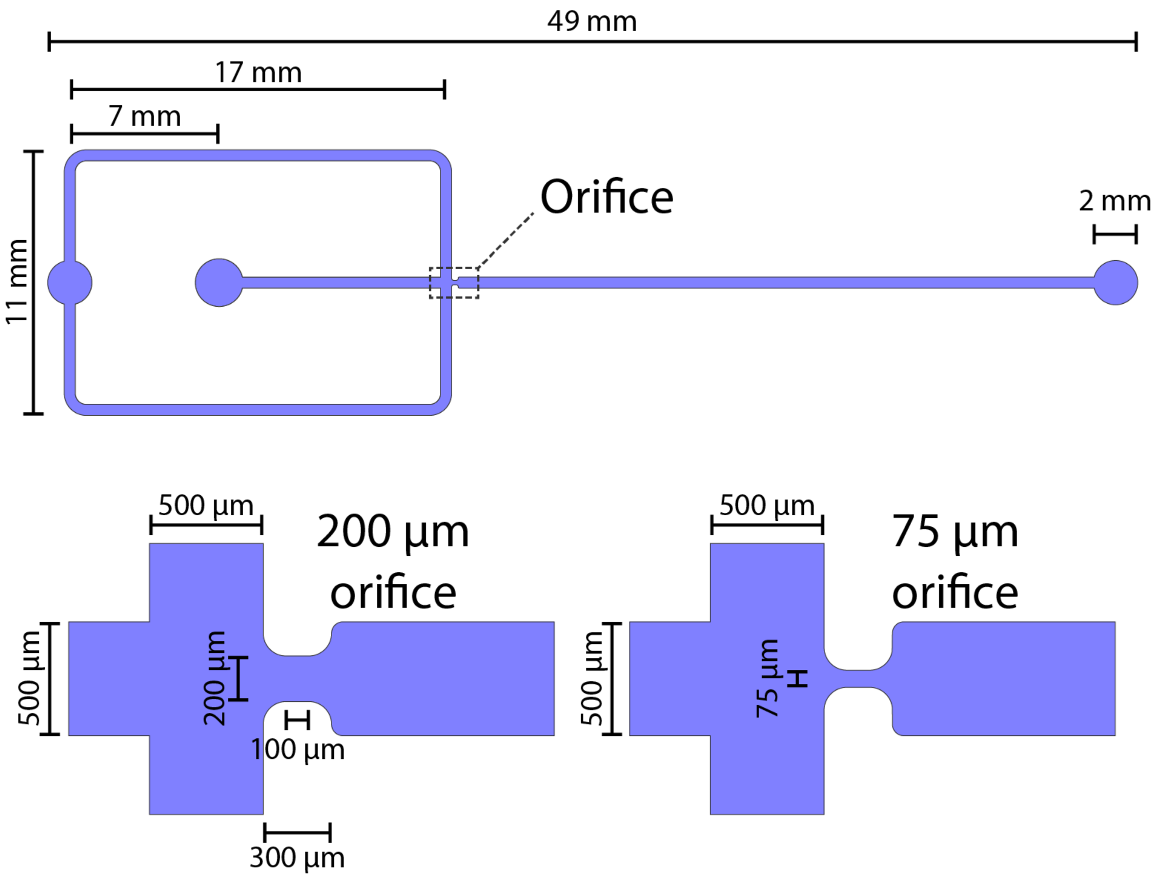

Figure S4: Dimensions of microfluidic systems used for droplet generation.

Supplementary Information S5: Advancement of water along the sidewalls of the water-treated PDMS channel

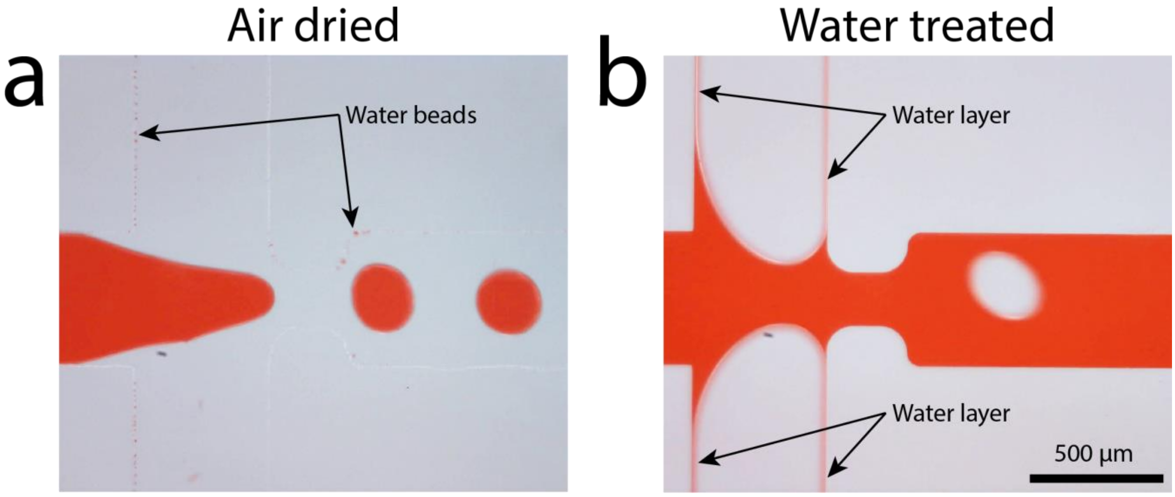

Figure S5: Comparison of water adhesion onto the sidewalls of air dried and water-treated PDMS channels: **(a)** Formation of water beads along the sidewalls of the air dried PDMS channel, showing the hydrophobic properties of the surface. **(b)** Advancement of water along the sidewalls of the water-treated PDMS channel, indicating the hydrophilic properties of the surface.

Supplementary Information S6: Droplet generation rate in the 'threading' mode

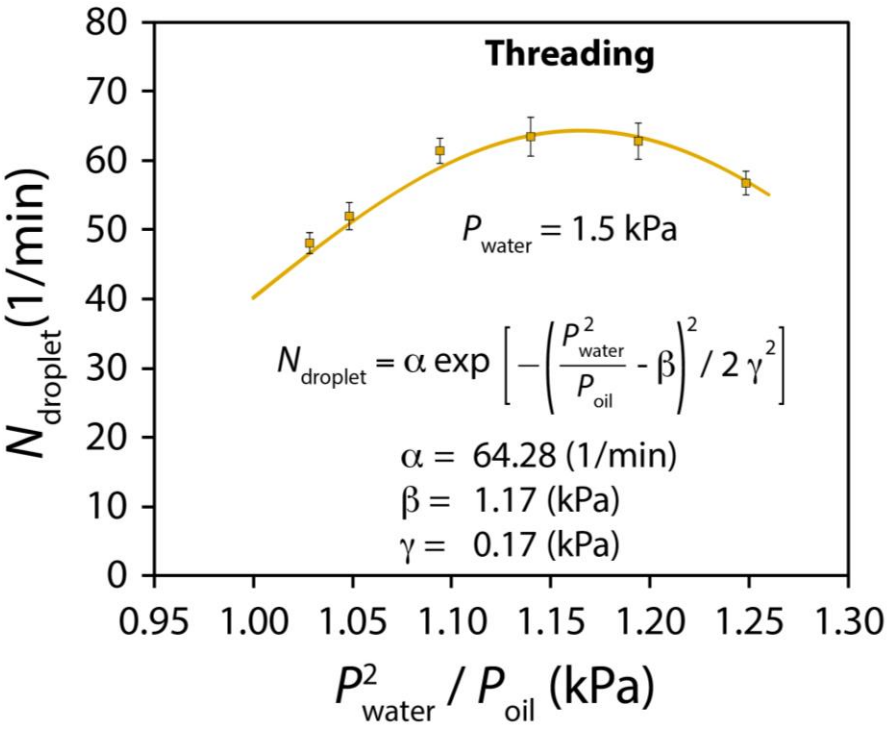

Figure S6: Variations of droplet generation rate ($N_{droplet}$) against P^2_{water}/P_{oil} in the 'threading' mode at P_{water} = 1.5 kPa. Average ± standard deviation values are based on 50 droplets per each experiment, with 4 experimental repeats for each device, and 3 devices being used for each experiment.

Supplementary Information S7: Asynchronous generation of oil droplets using a displacement (syringe) pump

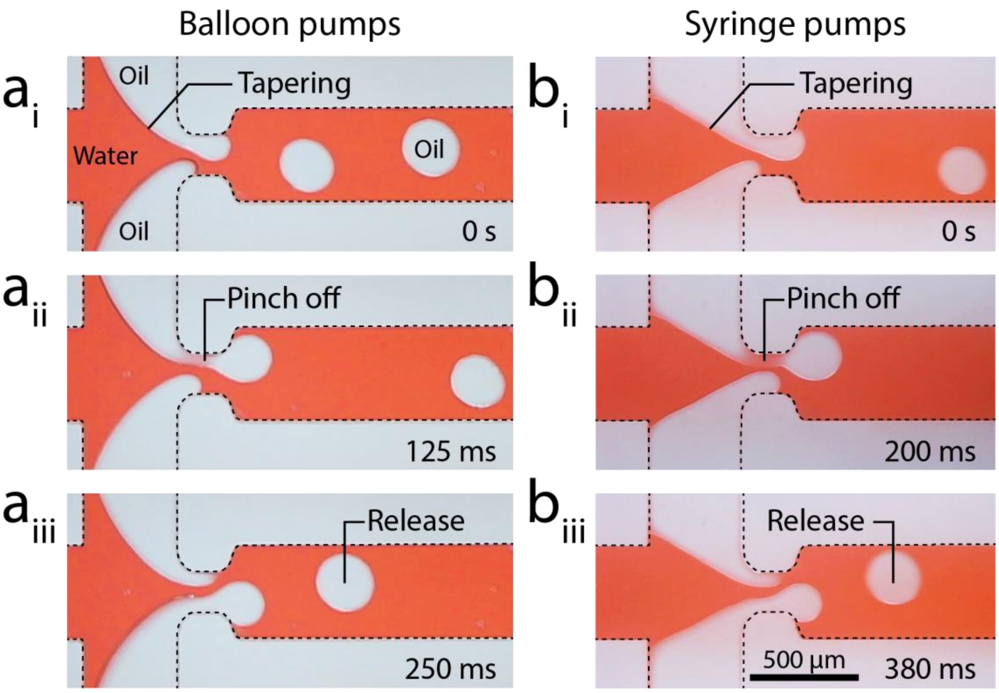

Figure S7: Asynchronous generation of oil droplets in dripping mode using: **(a)** Pressure pumps made of latex balloon operating at P_{water} ~1.59 kPa and P_{oil} ~2.38 kPa, and **(b)** Syringe pumps operating at Q_{water} = 25 µL/min, Q_{oil} = 0.5 µL/min.

Supplementary Information S8: Identification of droplet generation modes using syringe pumps

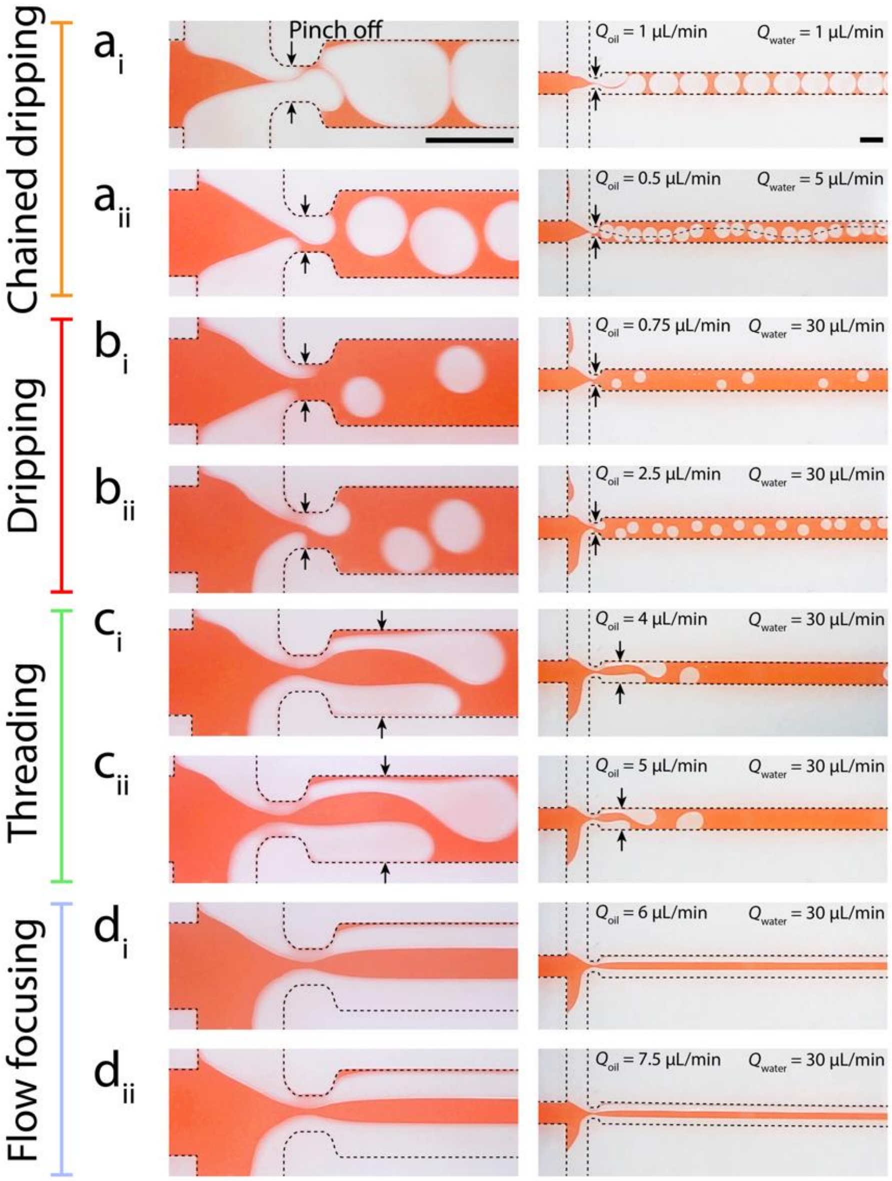

Figure S8: Identification of various droplet generation modes using syringe pumps: **(a)** ‘Chained dripping’ mode, **(b)** ‘Dripping’ mode, **(c)** ‘Threading’ mode. **(d)** ‘Flow focusing’ mode, which is only achievable using a syringe pump. Scale bars are 500 µm.

Supplementary Information S9: Asynchronous generation of oil droplets at various oil viscosities

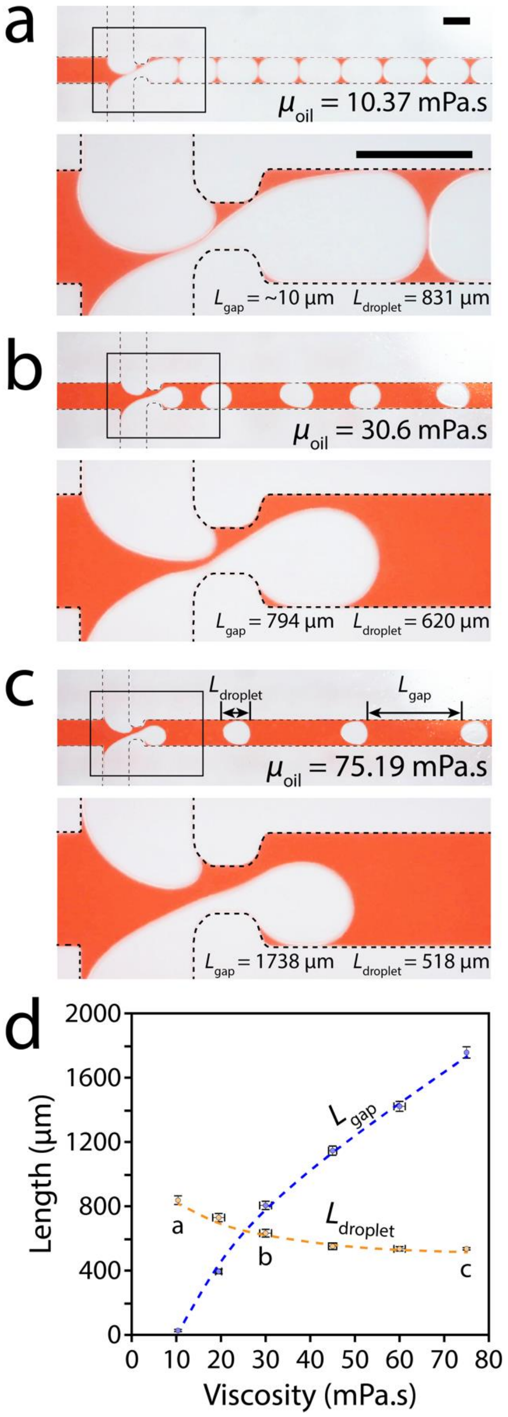

Figure S9: Studying the asynchronous generation of oil droplets at various oil viscosities: **(a-c)** Asynchronous generation of oil droplets at oil viscosities of 10.37, 30.6 and 75.19 mPa.s. **(d)** Variations of droplet size and gap against oil viscosity. Scale bars are 500 µm. Average ± standard deviation values are based on 50 droplets per each experiment, with 3 experimental repeats for each device, and 3 devices being used for each experiment.

Supplementary Information S10: Measuring the balloon inflation pressure and droplet size over long-term experiments

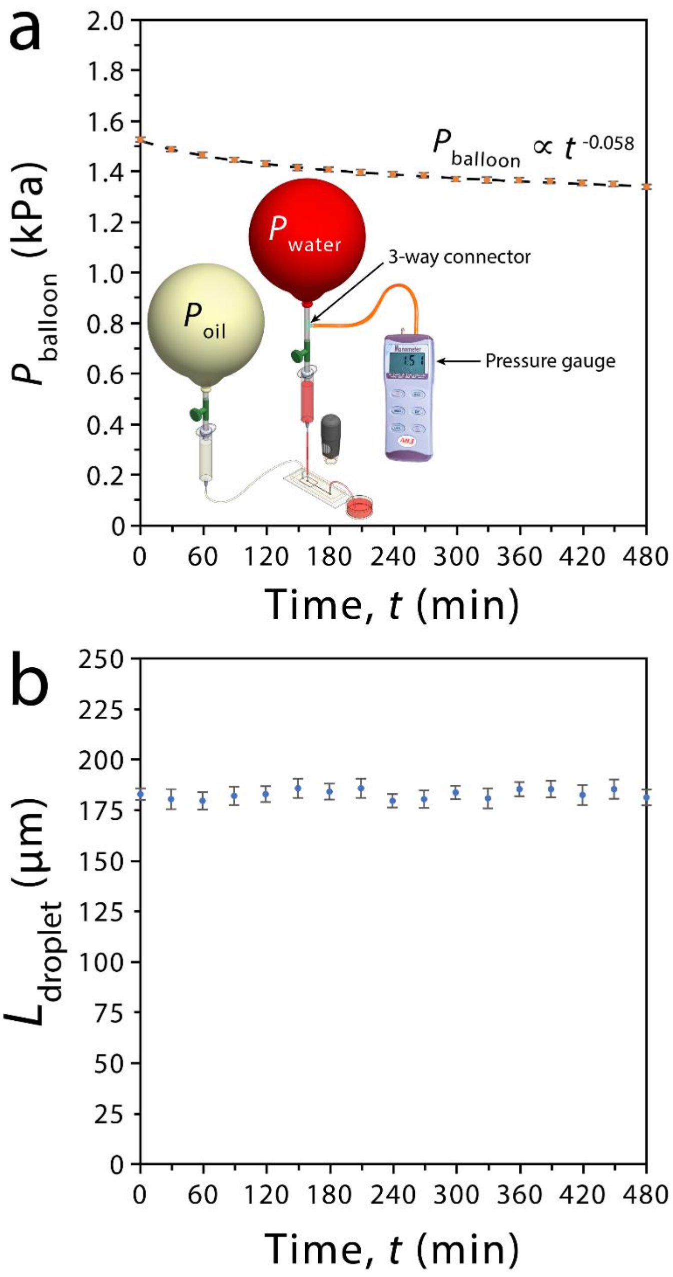

Figure S10: The stable generation of droplets over an extended time period. **(a)** Variations of balloon inflation pressure measured using a digital pressure gauge (AHJ Systems, 8205) over time. **(b)** Variations of droplet size over time. Average ± standard deviation values are based on 30 droplets per each experiment, with 3 experimental repeats for each device, and 3 devices being used in parallel for each experiment.

Supplementary Information S11: Mechanically clamped PDMS based microfluidic flow focusing system

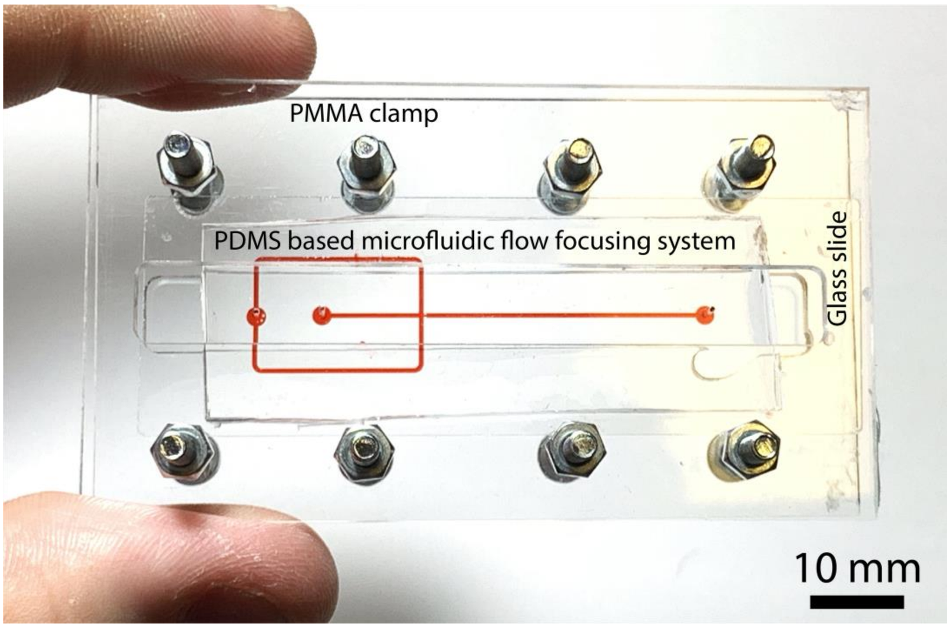

Figure S11: Mechanically clamped microfluidic system consisting of a clamp made of polymethyl methacrylate (PMMA), a PDMS based microfluidic flow focusing system for droplet generation, and a glass microscope slide. This is to stop leakage of liquid while avoiding plasma treatment. The channels were filled with red food dye for visualisation.

Supplementary Information S12: Variations of droplet size and gap in successive squeezing cycles.

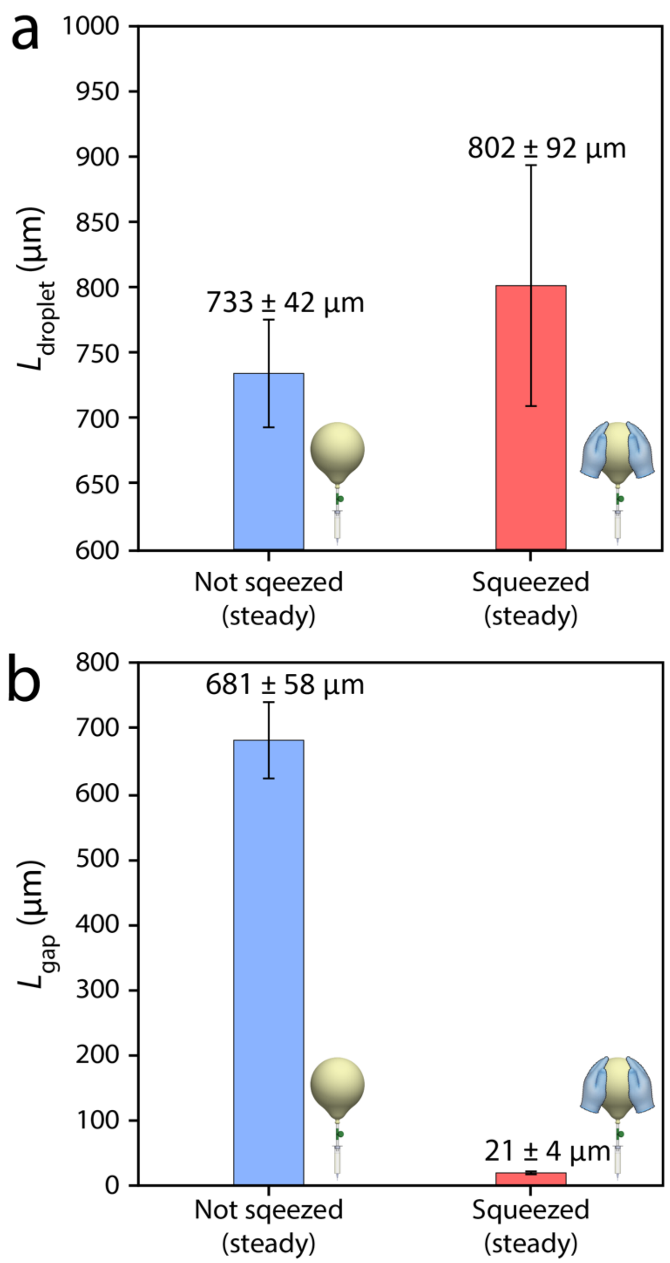

Figure S12: Variations of **(a)** Droplet length and **(b)** Droplet gap in successive squeezing cycles. Average ± standard deviation values are based on 5 cycles, with 6 repeats per each device, and 3 devices used in each experiment.

Supplementary Information S13: Schematics showing potential future applications

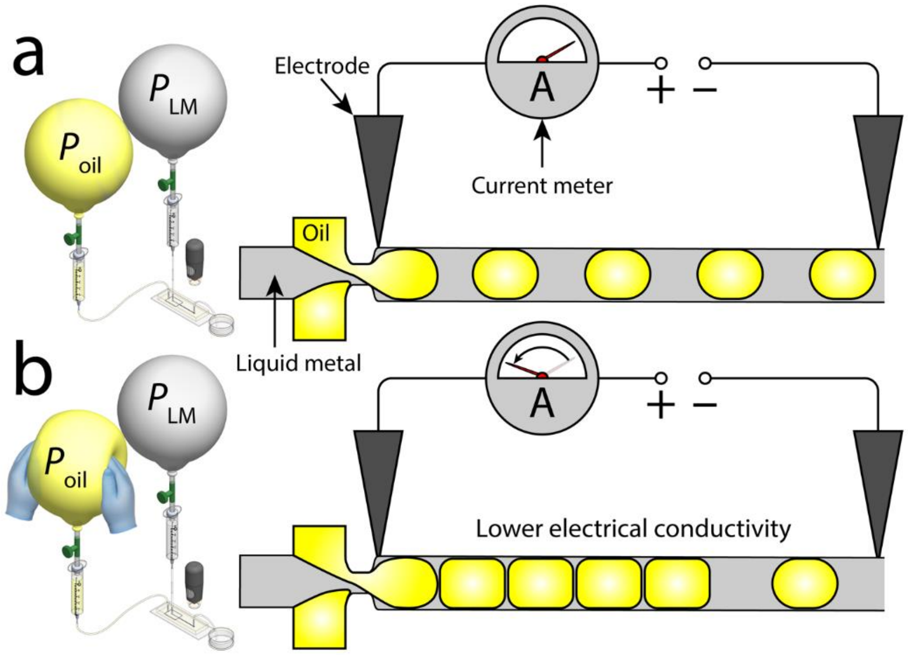

Figure S13a: Schematics showing the dynamic change of device electrical conductivity by varying the gap between the oil droplets using gallium based liquid metal alloys as the continuous phase and oil as the dispersed phase, providing opportunities for making reconfigurable soft electronic systems.

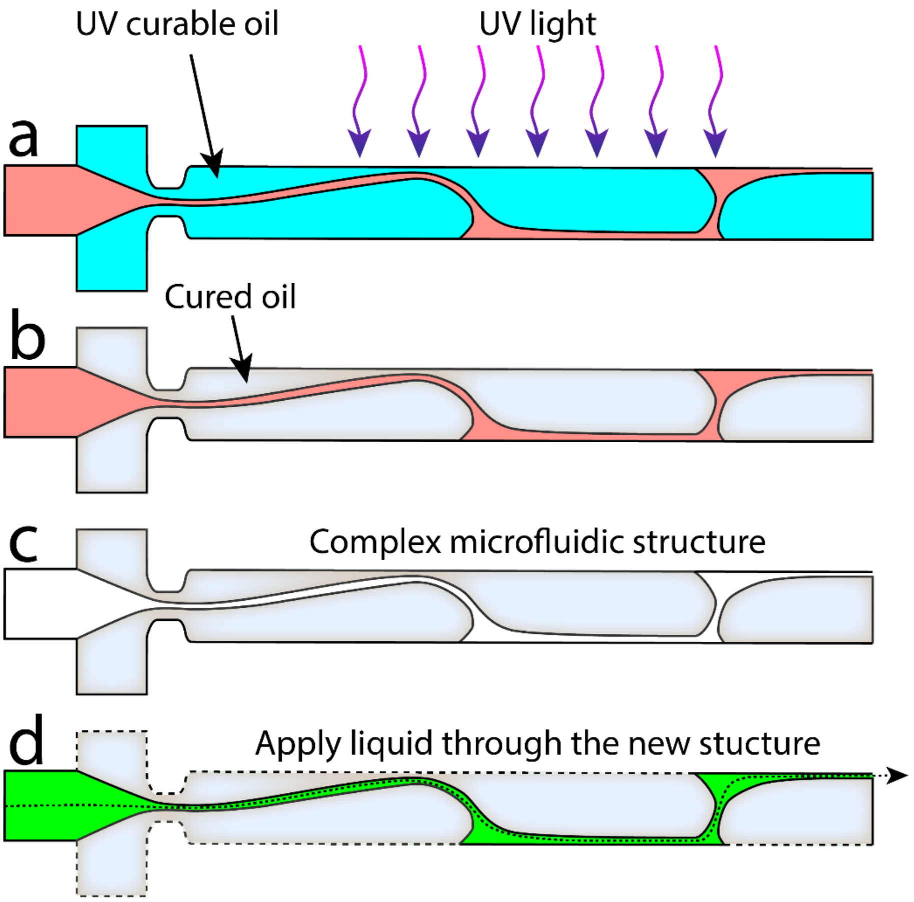

Figure S13b: Schematics showing the fabrication of complex microfluidic structures using UV curable oil.

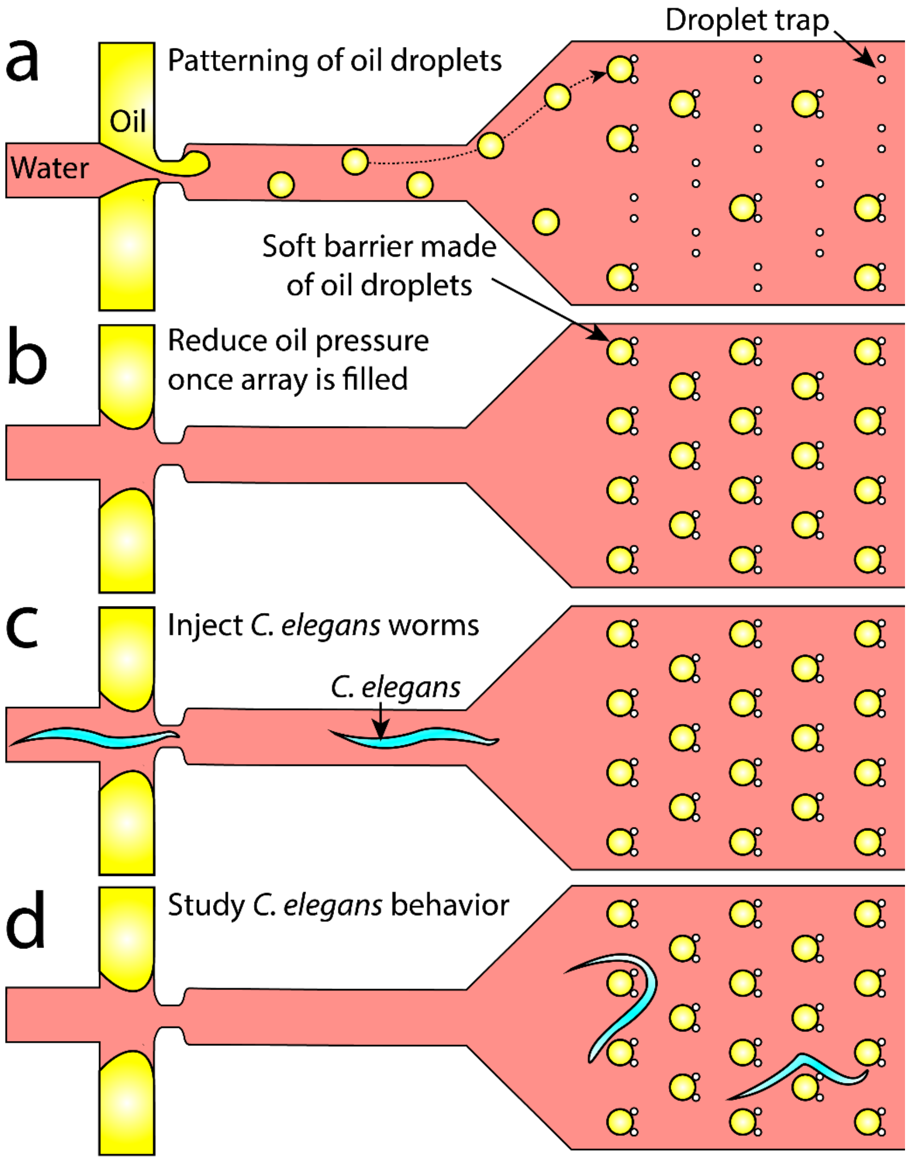

Figure S13c: Schematics showing the patterning of oil droplets using an array of micropillars to serve as soft barriers for studying the behaviour of multi-cellular organisms such as Caenorhabditis elegans worms.

Supplementary Information S14: Measuring the balloon inflation pressure using a digital pressure gauge

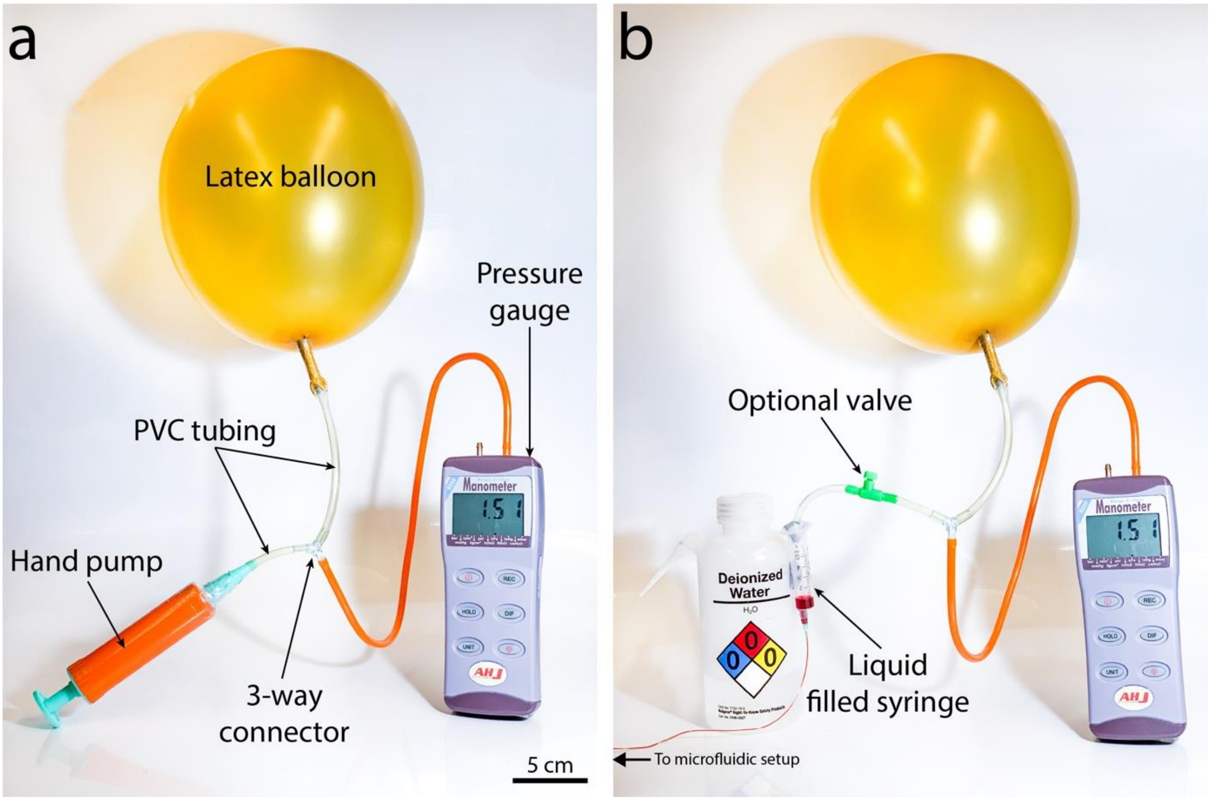

Figure S14: Measuring the balloon inflation pressure using a digital pressure gauge (AHJ Systems, 8205). **(a)** The inflation pressure can be measured while the balloon is inflated using a manual hand pump. **(b)** The inflation pressure can be measured in real-time while running experiments.
